# Optimizing TiO_2_/HfO_2_ Multilayer RRAM for Self-Rectifying Characteristics

**DOI:** 10.3390/mi17010049

**Published:** 2025-12-30

**Authors:** Chan-Hyeok Nam, Myung-Hyun Baek

**Affiliations:** 1 Department of Electronic Engineering, Gangneung-Wonju National University, Gangneung 25457, Republic of Korea; nch2007@naver.com; 2 Department of Electronic and Semiconductor Engineering, Gangneung-Wonju National University, Gangneung 25457, Republic of Korea

**Keywords:** neuromorphic systems, sneak current, selector layer, bilayer structure, work function

## Abstract

Sneak current refers to leakage currents in RRAM crossbar arrays without selector devices, disrupting the accuracy of weighted sum operations in neuromorphic systems, leading to performance degradation and increased power consumption. This study presents a bilayer RRAM structure with a selector layer designed to suppress sneak current in neuromorphic synapse arrays. By utilizing a TiO_2_/HfO_2_ bilayer structure, it is demonstrated that increasing the thickness of TiO_2_ and the work function of the top electrode effectively suppresses current under reverse bias compared to single-layer devices. The bilayer structure achieves rectification levels of 10 to 30 times higher than the single-layer configuration, while increasing the work function of the top electrode yields rectification improvements ranging from 10 to 40 times. This approach enhances the accuracy of synaptic weighted sum operations.

## 1. Introduction

**Figure 1:**
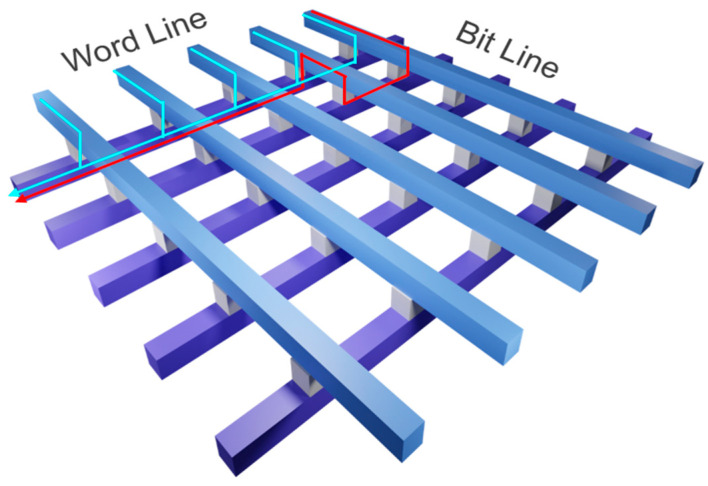
Schematic diagram of cross-point array. Blue lines show the desired current path, while red line shows sneak current path in synapse array.

Vector–matrix multiplication is a fundamental operation in deep learning algorithms, forming the basis of computations within neural network models. With the rapid advancement of deep learning algorithms, the optimization of this operation has gained significant importance. However, as deep learning models become increasingly complex, the computational demands associated with these operations have escalated dramatically. This often surpasses the processing capabilities of conventional hardware architectures, underscoring the need for more efficient and specialized computing solutions. To address this challenge, GPU-based AI computation has been employed. However, it demands substantial computational power. Consequently, research has shifted towards neuromorphic systems, which emulate the large-scale parallel connectivity of human brain neurons, with the goal of achieving efficient parallel computation while significantly reducing power consumption. Neuromorphic systems consist of neurons and their interconnecting synapses, where synapses function as the system’s memory units. Consequently, identifying appropriate memory devices for synaptic elements is of paramount importance. In this study, Resistive Random Access Memory (RRAM) is employed as a synaptic device. RRAM, characterized by its simple Metal-Insulator-Metal (MIM) structure and two-terminal operation, is particularly suitable for integration into large-scale memory arrays [1]. These attributes facilitate the efficient realization of high-density synaptic networks, a critical requirement for neuromorphic computing architectures. Large-scale integrated arrays are commonly organized in a cross-point array structure, as depicted in Figure 1. In this arrangement, Word Lines (WL) and Bit Lines (BL) intersect, with an RRAM device serving as a synaptic element at each intersection. This configuration supports the dense integration of memory devices, thereby enabling the development of large-scale neuromorphic systems. Ideally, the current should flow only from the word line (WL) to the selected bit line (BL). However, unintended current paths, referred to as sneak currents, can pass through unselected BLs. In cross-point arrays, such sneak currents often lead to system errors [2]. This issue mainly arises from the two-terminal structure of RRAM, which lacks inherent rectification. As a result, sneak current paths, involving at least one reverse-direction current, can emerge. This phenomenon presents a major obstacle to the accurate implementation of large-scale neuromorphic systems based on cross-point array architectures. To mitigate reverse currents, researchers have explored integrating additional components such as transistors or diodes into RRAM cells to create configurations like 1-Transistor-1-Resistor (1T1R) and 1-Diode-1-Resistor (1D1R) [3,4]. Although effective, these methods increase the overall array size due to the addition of extra elements. In contrast, the 1-Selector-1-Resistor (1S1R) structure provides a notable advantage. By incorporating a selector layer directly into the RRAM cell, it achieves rectifying functionality without substantially increasing the array’s area, thus preserving the high integration density of the memory devices [5]. To test this approach, we fabricated a bilayer RRAM structure using TiO_2_ as the selector layer, in place of the single-layer HfO_2_-based structure. Furthermore, by adjusting the work function of the top electrode, the band offset between the metal and the TiO_2_ selector layer can be increased. The resulting enlarged energy barrier under reverse-bias conditions strengthens the device’s rectifying behavior, working in conjunction with the selector to further suppress unwanted reverse currents in the cross-point array.

## 2. Architecture

**Figure 2:**
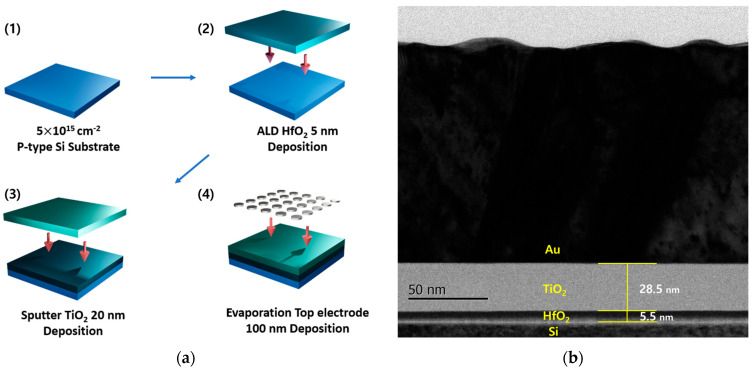
(**a**) Fabrication process of the proposed multilayer RRAM with self-rectifying characteristics and (**b**) Cross-sectional TEM image of the fabricated TiO_2_/HfO_2_ multilayer RRAM device.

To suppress sneak currents, we fabricated RRAM devices using two key strategies. The first approach involved the creation of a bilayer RRAM structure, utilizing TiO_2_ as a selector layer in conjunction with HfO_2_, rather than employing a single HfO_2_ layer [2,6]. This TiO_2_/HfO_2_ bilayer RRAM is designed to enhance the rectifying properties of the device [6,7]. The second approach focused on modifying the work function of the top electrode in the RRAM. We anticipated that, as the work function increases, the reverse current would decrease further. Figure 2 describes the fabrication process of the proposed RRAM device. First, a P-type Si substrate doped to a concentration of 5 × 10^15^ cm^−2^ was prepared. In this study, the Metal–Insulator–Semiconductor (MIS) structure was adopted primarily due to its high compatibility with standard CMOS fabrication processes. When using a silicon substrate directly or when integrating the process on a CMOS platform with poly-Si gates, the existing CMOS gate electrode can be utilized as the bottom electrode of the MIS-type RRAM device, providing a clear advantage for scalable integration [8]. Next, a 5 nm layer of HfO_2_ was deposited using the Atomic Layer Deposition (ALD) equipment at the Semiconductor Process Laboratory of Gangneung-Wonju National University (GWNU). The deposition was carried out by introducing the TEMAHf precursor and H_2_O reactant, with a purge time of 20 s for each cycle. Following this, a 20 nm layer of TiO_2_ was deposited via RF sputtering. TiO_2_ was selected as the selector material for several reasons. First, the conduction band offset (E_c_ offset) between TiO_2_ and HfO_2_ is sufficiently large, which enables effective suppression of reverse current. Second, under forward bias, the band offset between TiO_2_ and the bottom electrode (p-type Si) is minimal, facilitating efficient electron transport toward the top electrode. When deposited with sufficient thickness, TiO_2_ does not act as a switching layer but rather serves as a barrier layer, making it suitable for rectifying behavior in the bilayer structure [6]. Lastly, the top electrode was formed using E-beam evaporation with metals of varying work functions. Top electrode patterning and etching were also carried out using the mask aligner and dry etcher of the Semiconductor Process Laboratory at GWNU. Metals with low work functions, such as Al, Ti, and Cu, as well as a higher work function metal, Au, were deposited to a uniform thickness of 100 nm to evaluate their influence on the device’s performance. Al, Ti, Cu, and Au were selected as top electrodes because their work functions are distributed at relatively uniform intervals, allowing a systematic investigation of how incremental changes in work function influence the current asymmetry of the device. The corresponding work-function differences among these metals are summarized in Table 1, which further supports the rationale for selecting this specific set of electrode materials. In addition, a single-layer HfO_2_ RRAM was fabricated for comparison with the bilayer RRAM. This comparative analysis enabled us to evaluate the effectiveness of the bilayer structure in suppressing reverse currents, commonly referred to as sneak currents [9].

## 3. Measurement Results

### 3.1. Single-Layer RRAM Measurement

**Figure 3:**
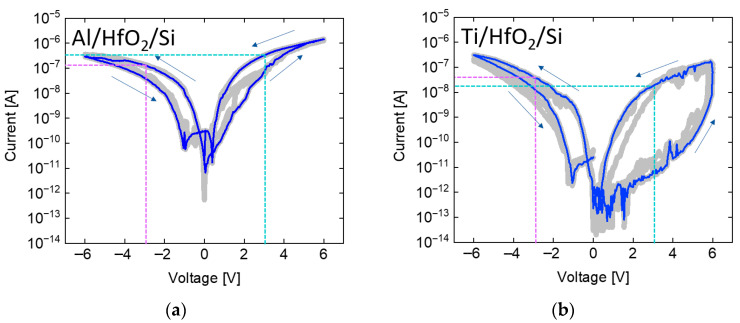

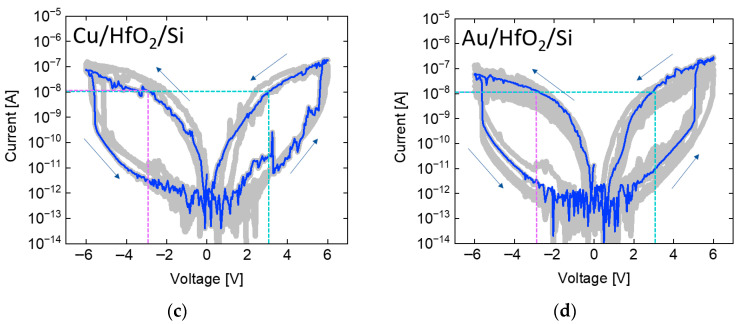
Measurement results of single-layer HfO_2_ RRAM with (**a**) aluminum, (**b**) titanium, (**c**) copper, and (**d**) gold electrodes. Reverse currents cannot be sufficiently suppressed regardless of the metal electrode.

To investigate the effect of various structures on suppressing sneak currents, a DC sweep measurement was performed on single-layer HfO_2_ RRAM devices. These measurements were conducted using the vacuum probe station in the Department of Electronics and Semiconductor Engineering at GWNU. The voltage range was set from 0 to 6 V, and for each stack with a different electrode material, five devices were measured, with each device tested 20 times. The repeated measurements are depicted as gray lines, and the most frequently observed current profile is highlighted by a blue line. The purple and sky blue dotted lines correspond to the read currents measured at the read voltages, respectively. A comparison of the read voltages at 3 V and −3 V was performed to determine whether the negative bias current exceeded or fell below the positive bias current. The read voltage of ±3 V was selected because the SET voltage is typically formed in the range of 4–5 V, and 3 V is sufficiently lower than this threshold to prevent unintended switching during read operations. This analysis was used to evaluate the effectiveness of sneak current suppression. The results presented in Figure 3 highlight the influence of the electrode work function on current flow in a single-layer HfO_2_ RRAM. The single-layer HfO_2_ RRAM devices incorporating Al, Ti, Cu, and Au top electrodes did not demonstrate meaningful self-rectifying behavior. Although the work function of these metals varies significantly, the measured current levels under positive and negative bias conditions were comparable across all cases. This indicates that the use of different electrode work functions in the single-layer configuration does not sufficiently alter the current conduction characteristics to enable rectification. As a result, the reverse current was not effectively suppressed, and the rectification ratio remained close to unity regardless of the electrode material. In summary, the results indicate that a synaptic array based on a single-layer HfO_2_ RRAM lacks self-rectifying behavior. Consequently, without further structural modifications, sneak current issues are likely to occur, potentially compromising the reliability of neuromorphic computing applications.

### 3.2. Multilayer RRAM Measurement

**Figure 4:**
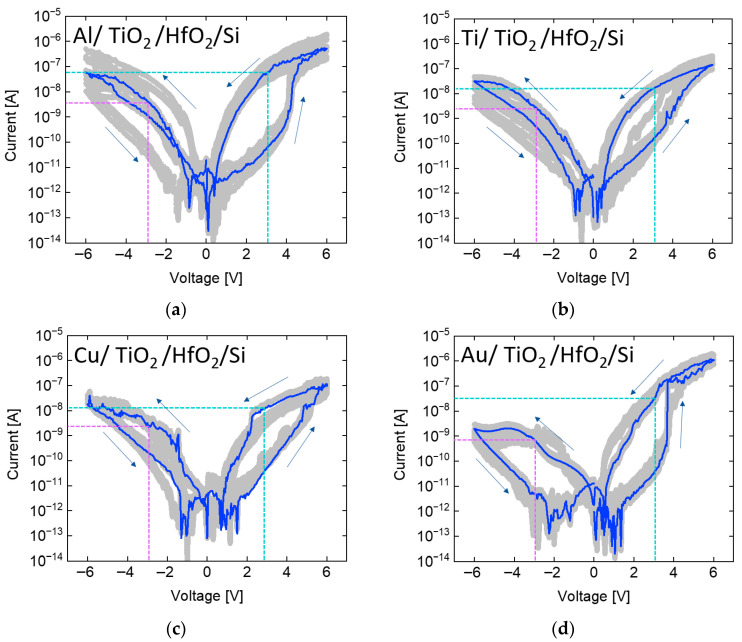
Measurement results of TiO_2_/HfO_2_ bilayer RRAM with (**a**) aluminum, (**b**) titanium, (**c**) copper, and (**d**) gold electrodes. Compared to the single-layer structure, reverse currents are suppressed by more than an order of magnitude.

Due to the inability of the single-layer HfO_2_ structure to effectively suppress sneak currents, a TiO_2_ film was introduced as a selector layer to create a TiO_2_/HfO_2_ bilayer RRAM. As shown in Figure 4, to verify the self-rectifying behavior of the bilayer structure, measurements were performed following the same procedure used for the single-layer devices, in which five devices per stack—each incorporating different electrode materials—were tested with 20 repeated measurements each. Subsequently, the influence of different electrode work functions on the reverse current was analyzed. Following the same procedure as that used for the single-layer RRAM, the repeated measurements are depicted as gray lines, and the most frequently observed current profile is highlighted by a blue line. The purple and sky blue dotted lines represent the read conditions. In the single-layer HfO_2_ RRAM with Al, the negative current was lower than the positive current; however, the difference was insufficient to indicate effective suppression of sneak currents. By contrast, the TiO_2_/HfO_2_ bilayer RRAM exhibited a more than tenfold difference in current, demonstrating pronounced self-rectifying behavior. Moreover, revealed that for Ti, which exhibited a higher negative current in the single-layer structure, the bilayer structure effectively suppressed the reverse current [6]. This result suggests that the introduction of the TiO_2_ layer significantly enhances current rectification [10]. Similarly, for the Cu and Au electrodes that exhibited nearly symmetric current levels under positive and negative biases in the single-layer structure, the bilayer RRAM effectively suppressed the reverse current [11]. Although Cu is known to operate through the Conductive Bridge Random Access Memory (CBRAM) mechanism—and is likely doing so in this study—the focus of this research lies not in distinguishing the switching mechanism (whether CBRAM or oxygen vacancy-based), but in evaluating the rectifying behavior. The rectifying characteristics are influenced by the thickness of the TiO_2_ layer, the conduction band offset between TiO_2_ and HfO_2_, and the work function of the top metal electrode. From this perspective, both switching mechanisms can be treated equivalently when analyzing self-rectifying behavior. In the subsequent measurement, Au—possessing the highest work function—exhibited the lowest negative current in the bilayer structure. This observation demonstrates that the bilayer configuration more effectively leverages work function differences to suppress sneak currents, highlighting its potential as a promising solution for realizing self-rectifying behavior [12]. Au, having the highest work function among the investigated metals, exhibits the strongest current asymmetry in this study. However, its practical use is limited by its high cost as a noble metal. In this work, Au was employed not for cost-efficient fabrication but to verify whether a sufficiently high work function directly enhances current asymmetry. For practical large-scale memory array implementation, alternative metals with comparable work functions but lower cost should be considered.

## 4. Discussion

### 4.1. Rectifying Mechanism of Bilayer RRAM

**Figure 5:**
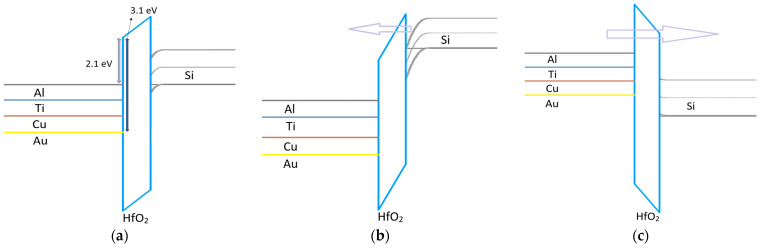
Energy band diagram of single-layer HfO_2_ RRAM under (**a**) zero bias, (**b**) forward voltage bias, and (**c**) reverse voltage bias. In both cases, electrons can easily penetrate the thin HfO_2_ barrier.

To understand why the bilayer RRAM demonstrates self-rectifying characteristics more effectively than the single-layer structure, the thickness and height of the electron-perceived barrier in the energy band under forward and reverse voltage conditions must be analyzed. Figure 5 illustrates the energy barrier under forward and reverse voltage conditions for the single-layer structure. As illustrated in Figure 5, in the single-layer RRAM, HfO_2_ alone acts as the insulating layer. Due to its extremely thin nature, tunneling occurs under both forward and reverse bias, enabling reverse current to flow smoothly. This inability to form an effective barrier in the single-layer structure explains its failure to adequately control sneak currents, emphasizing the critical role of the bilayer structure in achieving improved rectification [12,13].

**Figure 6:**
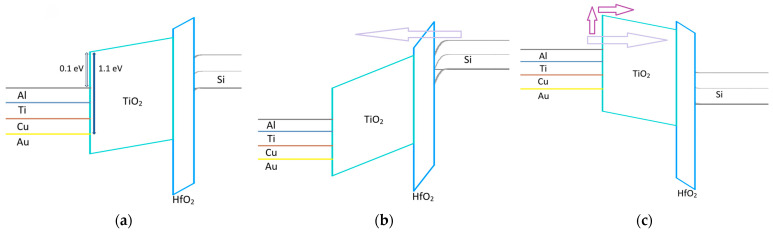
Energy band diagram of TiO_2_/HfO_2_ bilayer RRAM under (**a**) zero bias, (**b**) forward voltage bias, and (**c**) reverse voltage bias. Under reverse bias, electrons encounter the thick TiO_2_ barrier, which effectively mitigates reverse current.

The bilayer RRAM, however, operates through a distinct mechanism when forward and reverse voltages are applied. As depicted in Figure 6, under forward bias, the current flows through the thin HfO_2_ layer via tunneling, resembling the behavior observed in the single-layer RRAM. In contrast, under reverse bias, the thicker TiO_2_ layer impedes direct tunneling, resulting in rectifying behavior. Metals with lower work functions experience a thinner tunneling barrier due to Fowler-Nordheim (FN) tunneling and exhibit a smaller band offset, which may facilitate thermionic-field emission (TFE) [6,14,15]. Moreover, gradually increasing the work function of the metal electrode elevates the barrier height at the Metal-TiO_2_ interface, resulting in enhanced suppression of sneak currents [16]. This mechanism enhances self-rectifying characteristics, enabling more effective control and mitigation of unwanted current paths.

**Figure 7:**
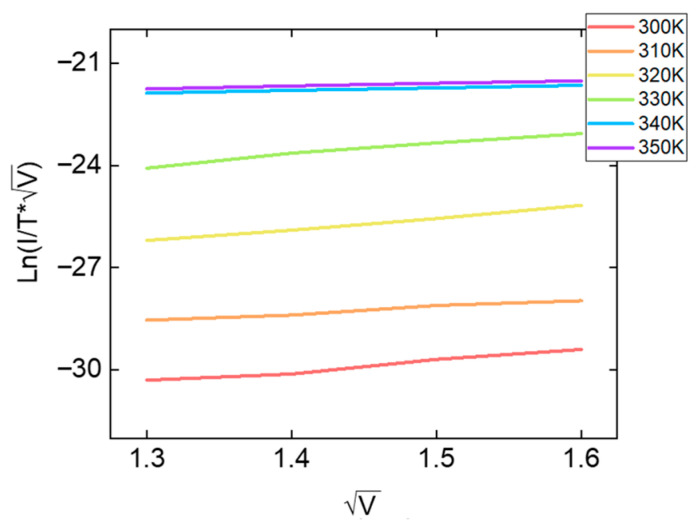
Temperature-dependent TFE plots of the TiO_2_/HfO_2_ bilayer RRAM device.

As shown in Figure 7, ln(J/(T√V)) exhibits a linear relation with √V for all temperatures from 300 K to 350 K. This linear behavior is a hallmark of TFE, indicating that carrier transport is dominated by temperature-assisted tunneling over a field-lowered barrier. The systematic shift of the curves with temperature further supports the TFE model, in which an increase in thermal energy reduces the effective barrier height, resulting in higher current levels.

### 4.2. TiO_2_ Thickness of Bilayer RRAM

**Figure 8:**
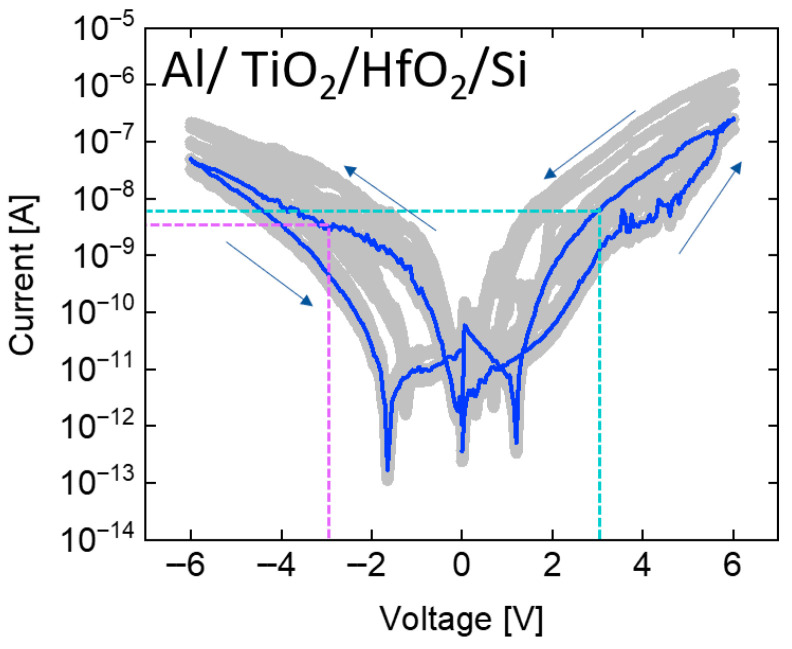
Measurement results of the TiO_2_/HfO_2_ bilayer RRAM with a 40 nm-thick TiO_2_.

In this study, the fabricated TiO_2_/HfO_2_ bilayer RRAM device incorporates a 20 nm-thick TiO_2_ layer. To evaluate whether this thickness is optimal, Figure 8 provides a meaningful comparison. Here, the gray lines represent the repeated measurement results, the blue line indicates the most frequently observed current profile, and the purple and sky blue dotted lines denote the currents measured at the read voltages. In the case of the device with a 40 nm-thick TiO_2_ layer, no SET operation is observed within 6 V, indicating poor suitability for low-voltage operation. For a TiO_2_ thickness of 20 nm, the transport behavior is well explained by TFE, as this thickness is sufficiently large for direct tunneling to be negligible. 

Thermionic-field emission equation:

(1) $$J\propto A^{*}T\sqrt{E}\cdot \exp\left(-\frac{q\left(\phi_{Β}-\Delta \phi \right)}{kT}\right),\;\Delta \phi =\sqrt{\frac{qE}{4\pi \varepsilon }}\;=\sqrt{\frac{qV}{4\pi \varepsilon d}}$$

According to the TFE model, the electric field E=V/d increases as the thickness d decreases, which in turn increases the image-force lowering Δϕ, reduces the effective barrier term, and significantly increases the current. For example, in the Al-electrode RRAM device, a TiO_2_ thickness of 20 nm yields a forward current of approximately 1 nA, whereas a hypothetical reduction to 15 nm would increase the current to about 3.8 nA. It should be noted that this value is not an experimental measurement, but an approximate analytical estimation based on the TFE model, included only to illustrate the expected degradation in rectifying behavior when the TiO_2_ thickness becomes excessively thin. This causes the rectification ratio to drop from approximately fifteen-to-one to roughly three-to-four-to-one, which likewise represents an estimated trend rather than an experimentally obtained value. Therefore, it can be concluded that a TiO_2_ thickness of 20 nm is optimal for achieving stable and reliable self-rectifying behavior in the bilayer RRAM structure.

### 4.3. Rectifying Ratio of Bilayer RRAM

**Figure 9:**
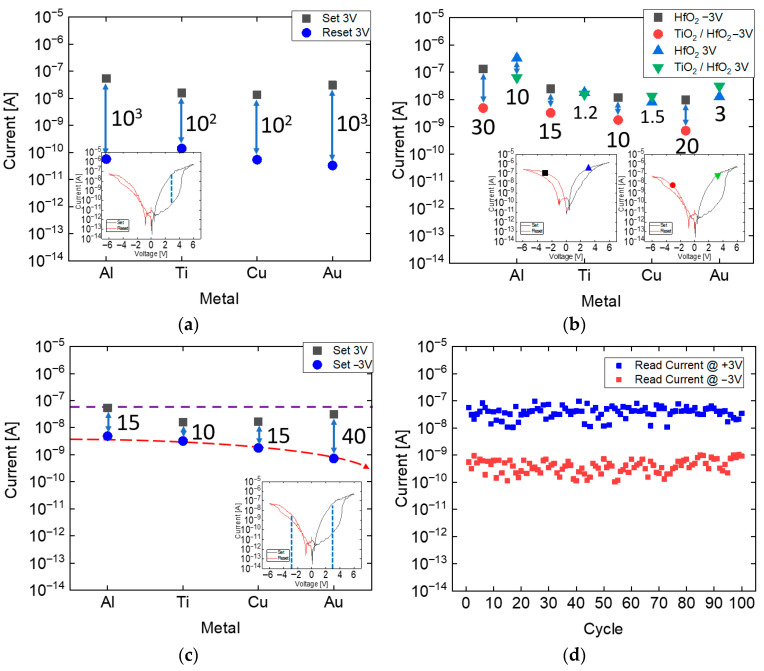
(**a**) ON/OFF ratio of the TiO_2_/HfO_2_ bilayer RRAM for different top electrodes, evaluated at a read voltage of +3 V, where the associated current ratios are labeled accordingly, (**b**) Comparison of the read currents of single-layer HfO_2_ RRAM and TiO_2_/HfO_2_ bilayer RRAM measured at +3 V and −3 V for different top electrodes, (**c**) Rectifying ratio of the TiO_2_/HfO_2_ bilayer RRAM, defined as the ratio of the read currents at +3 V and −3 V for different top electrodes and (**d**) Read currents at ±3 V in the SET state of TiO_2_/HfO_2_ bilayer RRAM.

After confirming that the bilayer RRAM exhibits significantly enhanced self-rectifying characteristics compared to the single-layer RRAM, an in-depth analysis of the memory window and the exact rectifying ratio of the bilayer structure was conducted using five devices per stack, each incorporating different electrode materials. Figure 9a depicts the memory window of the TiO_2_/HfO_2_ bilayer RRAM, highlighting a distinct separation between the Low Resistive State (LRS) and the High Resistive State (HRS). The resistance difference between these states is measured to be at least 10^2^ times, as shown in the graph. However, in the Al-electrode device, the HRS and LRS curves appear to overlap near approximately −2 V in the negative-bias region because the device exhibits rectifying behavior. In the single-layer Al RRAM, the HRS and LRS curves do not overlap, whereas in the bilayer structure this overlap becomes evident. This is attributed to the fact that, in the LRS, the bilayer device shows strong rectification: under reverse-bias conditions, the HfO_2_ layer alone is sufficiently thin for current to pass, but the additional TiO_2_ selector layer introduces a significant barrier, thereby suppressing the reverse current. Consequently, the observed overlap in the negative-bias region is unrelated to the memory window and does not contradict the non-volatile switching characteristics presented in Figure 9a. This substantial disparity in resistance levels ensures the clear differentiation of memory states, confirming the device’s reliability and its potential for non-volatile memory applications. Figure 9b,c provide a comparative analysis of the rectifying ratios for single-layer and bilayer RRAM structures. Figure 9b shows the forward (+3 V) and reverse (−3 V) current values measured in the SET state for both the single-layer and bilayer RRAM devices. In particular, the current ratio at −3 V is more than ten times larger in the bilayer structure compared to the single-layer device [17]. Figure 9c presents the current values measured at ±3 V in the SET state for the bilayer RRAM structure. The data show that as the work function of the top electrode increases, the reverse current decreases, as illustrated by the red trend line. In cross-point array operations, sneak current arises from reverse current paths between selected and unselected cells. For a selected cell, the WL is typically held at a high voltage while the BL is grounded. In contrast, unselected cells may experience an inverse condition—WL at ground and BL at high voltage—resulting in an effective reverse bias of −3 V across those cells. Thus, the current ratio at +3 V and −3 V serves as a practical definition of the rectifying ratio. This context supports the observation that the Au electrode, which possesses the highest work function among the tested metals, achieves a rectification ratio as high as approximately forty to one. In addition, Figure 9d presents the read current values measured at ±3 V in the SET state. The results indicate that applying ±3 V repeatedly does not significantly disturb the resistance states of the device, suggesting that the selected read voltages are appropriate for non-destructive operation. Rectifying ratios were also evaluated at ±2 V in addition to ±3 V. In the case of Au, the ratio slightly decreased from approximately forty to one at ±3 V to about thirty-five to one at ±2 V. Nevertheless, the device maintained meaningful self-rectifying behavior even under the lower read voltage condition. These findings highlight the importance of the bilayer configuration and the role of electrode work function engineering in enhancing rectifying behavior. The ability to modulate rectification through electrode selection further demonstrates the promise of the proposed bilayer RRAM, as summarized in Table 2, in mitigating sneak currents and improving performance in memory applications [18,19,20,21].

## 5. Conclusions

This study examined the mechanisms underlying sneak current suppression in single-layer and bilayer RRAM structures. While the single-layer HfO_2_ RRAM failed to exhibit self-rectifying behavior due to insufficient reverse current control, the TiO_2_/HfO_2_ bilayer RRAM demonstrated significantly enhanced rectification, achieving up to a 30-fold reduction in reverse current. Additionally, increasing the work function of the top electrode further improved the rectification ratio by up to 40 times. These results highlight the essential role of the bilayer structure and electrode engineering in enabling reliable memory operation and effective self-rectification. Importantly, the improved rectifying behavior and suppression of sneak currents facilitate accurate weighted-sum operations in neuromorphic synaptic arrays. This also suggests strong potential for energy-efficient, scalable integration in cross-point architectures, making the proposed bilayer RRAM structure a promising candidate for neuromorphic computing applications.

## Figures and Tables

**Table 1:** Top electrode work functions.

Al	Ti	Cu	Au
4.1 eV	4.3 eV	4.7 eV	5.1 eV

**Table 2:** Benchmark comparison of recent state-of-the-art RRAM devices.

	This Work	[12]	[19]	[22]
Rectifying Ratio	40	65	60	10^4^
CMOS Compatibility	◎	△	△	○
Operation Voltage	±3.7	±2.8	±4.75	±1.8
Read Power Consumption	370 nW	128.8 µW	47.5 nW	180 µW

## Data Availability

The original contributions presented in this study are included in the article. Further inquiries can be directed to the corresponding author.
